# Enhanced neutrophil extracellular trap formation in COVID-19 is inhibited by the protein kinase C inhibitor ruboxistaurin

**DOI:** 10.1183/23120541.00596-2021

**Published:** 2022-04-04

**Authors:** Rebecca Dowey, Joby Cole, A.A. Roger Thompson, Rebecca C. Hull, Chenghao Huang, Jacob Whatmore, Ahmed Iqbal, Kirsty L. Bradley, Joanne McKenzie, Allan Lawrie, Alison M. Condliffe, Endre Kiss-Toth, Ian Sabroe, Lynne R. Prince

**Affiliations:** 1 Dept of Infection, Immunity and Cardiovascular Disease, University of Sheffield, Sheffield, UK; 2 Sheffield Teaching Hospitals NHS Foundation Trust, Sheffield, UK; 3 Faculty of Medicine, Dentistry and Health, University of Sheffield, Sheffield, UK; 4 Dept of Oncology and Metabolism, University of Sheffield, Sheffield, UK

## Abstract

**Background:**

Neutrophil extracellular traps (NETs) are web-like DNA and protein lattices which are expelled by neutrophils to trap and kill pathogens, but which cause significant damage to the host tissue. NETs have emerged as critical mediators of lung damage, inflammation and thrombosis in coronavirus disease 2019 (COVID-19) and other diseases, but there are no therapeutics to prevent or reduce NETs that are available to patients.

**Methods:**

Neutrophils were isolated from healthy volunteers (n=9) and hospitalised patients with COVID-19 at the acute stage (n=39) and again at 3–4 months post-acute sampling (n=7). NETosis was measured by SYTOX green assays.

**Results:**

Here, we show that neutrophils isolated from hospitalised patients with COVID-19 produce significantly more NETs in response to lipopolysaccharide (LPS) compared to cells from healthy control subjects. A subset of patients was captured at follow-up clinics (3–4 months post-acute sampling), and while LPS-induced NET formation is significantly lower at this time point, it remains elevated compared to healthy controls. LPS- and phorbol myristate acetate (PMA)-induced NETs were significantly inhibited by the protein kinase C (PKC) inhibitor ruboxistaurin. Ruboxistaurin-mediated inhibition of NETs in healthy neutrophils reduces NET-induced epithelial cell death.

**Conclusion:**

Our findings suggest ruboxistaurin could reduce proinflammatory and tissue-damaging consequences of neutrophils during disease, and since it has completed phase III trials for other indications without safety concerns, it is a promising and novel therapeutic strategy for COVID-19.

## Abstract 2

**Proinflammatory neutrophil extracellular traps are increased during acute #COVID19 and can be reduced by the protein kinase C inhibitor and orally active drug, ruboxistaurin.** https://bit.ly/3nH6sxD

## Introduction

Excessive inflammation is characteristic of severe coronavirus disease 2019 (COVID-19). Neutrophils are recruited to the lungs in response to severe acute respiratory syndrome coronavirus 2 (SARS-CoV-2) infection and are a principal cause of tissue damage and ongoing inflammation [1]. Neutrophil activation at the alveolar space is thought to contribute to the development of acute respiratory distress syndrome (ARDS) in COVID-19 as well as in other lung infections [2–4]. Here, neutrophils perform antimicrobial effector functions including production of reactive oxygen species (ROS), degranulation of cytotoxic proteins and release of neutrophil extracellular traps (NETs) *via* NETosis. NETs are extracellular DNA lattices coated in histones and antimicrobial proteins including cathepsins and myeloperoxidase (MPO). NETs are antimicrobial, but they also cause significant host tissue damage and exacerbate inflammation in multiple acute and chronic diseases, including those of the lung [5]. NET production is increased during COVID-19, with NETs identified in plasma, lung autopsy samples from deceased patients with COVID-19 and in bronchoalveolar lavage fluid (BALF) [6–11]. Furthermore, SARS-CoV-2 directly induces NETosis *in vitro*, *via* a ROS-dependent mechanism, and circulating markers of NETosis (including cell free DNA and neutrophil elastase (NE)) are associated with increased COVID-19 severity [6, 7, 12, 13]. NETs are highly pro-thrombotic *in vivo*, aggregating with platelets and the activated endothelium in COVID-19 to form microthrombi, which occlude the vasculature and further perpetuate inflammation [6]. Furthermore, SARS-CoV-2-induced NETs induce epithelial cell death, driving the catastrophic damage to the airway epithelium that is associated with severe disease [8]. This growing evidence indicates that inhibiting NET formation is an important and viable therapeutic strategy. Here we show for the first time that NETs are elevated in response to lipopolysaccharide (LPS) from neutrophils isolated from hospitalised patients with COVID-19 and that the orally active protein kinase C (PKC) inhibitor, ruboxistaurin (LY-333531), is a potent inhibitor of NETosis in this cohort. Since ruboxistaurin has completed phase III trials for other indications and is safe in humans, we believe it could be quick to enter the clinic as a new drug for COVID-19.

## Material and methods

### Human samples

Hospitalised patients with COVID-19 (n=39) admitted to the Royal Hallamshire Hospital, Sheffield, UK were recruited to the study and provided fully informed consent *via* The Sheffield Teaching Hospitals Observational Study of Patients with Pulmonary Hypertension, Cardiovascular and other Respiratory Diseases (STH-ObS) (REC 18/YH/0441, IRAS 248890, project title: Establishing the magnitude, breadth and durability of SARS-CoV-2 induced activation of innate immune blood cells (COVID-19 INNATE)). Ethical approval was given by the Yorkshire & The Humber – Sheffield Research Ethics Committee. Specific project approval was given by the STH-ObS Scientific Advisory Board. All patients had SARS-CoV-2 infection as confirmed by a PCR test. Peripheral blood samples were obtained mean±sd 3±3.1 days post hospital admission. Peripheral blood samples were also obtained for seven participants 3–4 months post-acute sampling. These individuals were not selected and instead were those who attended follow-up clinics during our study period. Anonymised clinical information was collected for all patients (see table 1 for all patient information and table 2 for follow-up patient data). Blood from healthy volunteers was taken according to the protocol: the control of innate immunity, host–pathogen interactions and leukocyte function in healthy volunteers (REC 05/Q2305/4, STH13927). Ethical approval was given by the Yorkshire & The Humber – Sheffield Research Ethics Committee. All participant information was anonymised, and subjects were identified by a unique number.

**TABLE 1:** All coronavirus disease 2019 (COVID-19) patient characteristics

**Demographics**	
Total number of participants	39
Age years (mean±sd)	57.4±12.3
Age years (range)	29–83
Female	12 (30.8)
Male	27 (69.2)
**Clinical data**	
Days following symptom onset of neutrophil sampling (mean±sd)	12.9±7
Days following symptom onset of neutrophil sampling (range)	4–43
Length of stay in hospital days (mean±sd)	11±13.2
WHO symptom severity score (mode)	1
Required supplemental oxygen	38 (97.4)
Received dexamethasone	32 (82.1)
Received tocilizumab	1 (2.6)
Admitted to ITU	3 (7.7)
Deaths	2 (5.1)
Neutrophil count ×10^9^ per L (mean±sd)	5.6±2.3
CRP mg·L^−1^ (mean±sd)	49±42.6
Platelet count ×10^9^ per L (mean±sd)	280±103
**Comorbidities**	
None	6 (15.4)
Diabetes (including pre-diabetes)	14 (35.8)
Hypertension	11 (28.2)
Asthma	8 (20.5)
Cancer	6 (15.4)
Cardiovascular disease	6 (15.4)
Obesity	4 (10.3)
Kidney disease	2 (5.1)
COPD	2 (5.1)
Bronchiectasis	1 (2.6)

Data are presented as n (%) unless otherwise stated. WHO: World Health Organization; ITU: intensive care unit; CRP: C-reactive protein.

**TABLE 2:** Follow-up patient characteristics

**Demographics**	
Total number of participants	7
Age years (mean±sd)	57.2±13.3
Age years (range)	29–70
Female	2 (28.5)
Male	5 (71.5)
Previous WHO symptom severity score (mode)	1
**Comorbidities**	
None	2 (28.6)
Diabetes (including pre-diabetes)	1 (14.2)
Asthma	1 (14.2)
Cardiovascular disease	1 (14.2)
Cancer	1 (14.2)
Obesity	1 (14.2)

Data are presented as n (%) unless otherwise stated. WHO: World Health Organization.

### Neutrophil isolation

Anti-coagulated blood (10 mL) (1.8 mg·mL^−1^ EDTA) was processed immediately after phlebotomy and functional studies carried out on freshly isolated cells. Neutrophils were isolated using EasySep™ Direct Human Neutrophil Isolation Kit (Stemcell technologies) as per manufacturer instructions. Mean±sd neutrophil yield for patient samples was 4.09×10^6^±1.9 cells·mL^−1^ whole blood.

### SYTOX green NET assays

Neutrophils were resuspended in RPMI 1640 (without phenol red) and 10 mM HEPES (Thermo Fisher, Waltham, MA, USA), and seeded (5×10^4^) in quadruplicate in a Nunc™ MicroWell™ 96-well flat bottom plate (Thermo Fisher). Cells were pre-incubated with either 200 nM ruboxistaurin (Selleckchem, Houston, TX, USA), 10 µM diphenyleneiodonium (DPI) (Cayman Chemical, Ann Arbor, MI, USA) or 10 µM dexamethasone (Sigma-Aldrich, St Louis, MO, USA) for 1 h (37°C, 5% CO_2_) before stimulation with either LPS (5 µg·mL^−1^) (*Escherichia coli* O111:B4) (Sigma-Aldrich), phorbol myristate acetate (PMA) (100 nM) (Sigma-Aldrich) or DMSO control. Neutrophils were incubated for a further 3 h before adding SYTOX™ Green nucleic acid stain (555 nM) (Thermo Fisher) to all wells, to measure extracellular DNA as a surrogate of NET formation. Extracellular DNA was quantified using a fluorescent plate reader (excitation/emission 490/537 nM) and median fluorescence values were reported.

### Immunocytochemistry

Neutrophils (5×10^5^) were seeded into IBIDI™ µ-slide 8-well chamber slides, pre-incubated for 1 h with ruboxistaurin (200 nM), and NETosis was induced by LPS and PMA as described above. Cells were fixed with 4% paraformaldehyde (PFA) for 15 min at room temperature. Wells were blocked and permeabilised with buffer containing 5% bovine serum albumin (BSA), 0.1% saponin and 5% normal goat serum, and then stained with a rabbit anti-MPO (A0398) primary antibody for 90 min at 37°C. A secondary goat anti-rabbit Alexa Fluor® 594 antibody (ab150088) was added for 45 min at 37°C. ProLong™ Gold Antifade Mountant with 4′,6-diamidino-2-phenylindole (DAPI) (Thermo Fisher) was used to stain DNA. Samples were imaged using a NIKON Widefield fluorescence microscope, using the 40× oil immersion objective lens. The DAPI (excitation/emission 395/455 nM) and Texas Red (excitation/emission 555/605 nM) filter sets were used for fluorescent imaging. Images were constructed using FIJI image analysis software, and the background was subtracted for the DAPI channel using FiJI.

### Human bronchial epithelial cell culture

Human bronchial epithelial cells (HBEC3-KT) were grown in a humidified incubator at 37°C, 5% CO_2_. Cells were maintained in basal growth medium; Keratinocyte-SFM (1X) with L-glutamine (Thermo Fisher), supplemented with bovine pituitary extract, epidermal growth factor and Gentamicin sulfate-Amphotericin – 1000 (GA-1000) (Lonza, Basel, Switzerland). Cells were passaged twice weekly when at 70–80% confluency and used for experiments between passage 12 and passage 22.

### Cell viability assay

Neutrophils (2.5×10^6^) were seeded in microcentrifuge tubes and stimulated to induce NET formation with PMA±ruboxistaurin as described above. Cells were spun at 2500 *g* for 5 min and the cell-free supernatants (SPN) were removed and stored at −80°C until required. HBEC3-KT cells were seeded into a 24-well plate at 1.2×10^6^ per plate and grown overnight to reach 90–100% confluency, before overnight incubation in basal media with depleted growth factors. HBEC3-KT cells were incubated with neutrophil SPNs at 1:2 dilution±ruboxistaurin (200 nM) for 24 h. CellTiter-Glo® was used as a measure of cell viability. Spent media was removed and pre-prepared CellTiter-Glo® reagent added at a 1:2 dilution with basal medium to the tissue culture plate. The plate was incubated (with shaking) at room temperature for 2 min then for an additional 10 min at room temperature (without shaking). Samples were added in duplicate to a white opaque 96-well plate (Costar, Washington, DC, USA) and luminescence determined using a fluorescent plate reader at 480 nm.

### Statistics

Data were plotted and analysed using GraphPad Prism version 9.2. A Shapiro–Wilk normality test was conducted on these data, where n=≥6 to determine the use of parametric and non-parametric analyses. Owing to missing values, a mixed-effect analysis with a Šidák post-test was used for comparing NET formation in acute COVID-19 patients with healthy controls. A one-tailed paired t-test was used for comparing NETosis in matched COVID-19 patients at the acute and follow-up stage. A one-tailed Wilcoxon matched-pairs signed rank test was used for the DPI inhibition data and a two-tailed Wilcoxon matched-pairs signed rank test was used for the ruboxistaurin inhibition data. A t-test was used for the epithelial cell viability data and for comparing NET formation in healthy controls and follow-up patients.

## Results

### Neutrophils isolated from patients with COVID-19 generate more NETs in response to LPS

**FIGURE 1:**
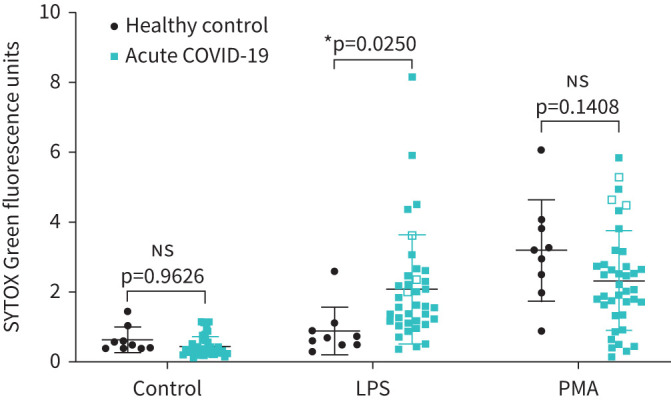
LPS-stimulated neutrophil extracellular trap (NET) release is significantly elevated in acute coronavirus disease 2019 (COVID-19) patients. Neutrophils isolated from peripheral whole blood from healthy control subjects (circles, n=9) or hospitalised patients with COVID-19 (squares, n=37 LPS, n=39 PMA) were stimulated for 3 h with either LPS (5 µg·mL^−1^) or phorbol myristate acetate (PMA) (100 nM). Open squares indicate patients (n=3) who were admitted to an intensive care unit during the study. SYTOX Green was added, and extracellular DNA release (NETs) was quantified using a fluorescent plate reader. A significant increase in NET formation was shown in acute COVID-19 patients in response to LPS but not in response to PMA. Statistical analysis used a mixed-effects model with a Šidák post-test. Error bars represent sd. ns: nonsignificant. *: p<0.05.

Neutrophils were isolated from venous blood from healthy volunteers (healthy controls) or patients hospitalised following a positive PCR test for SARS-CoV-2 (n=39). Of the 39 COVID-19 patients recruited to the study, 38 required supplemental O_2_, 32 received dexamethasone, 3 were subsequently admitted to intensive care and 2 died (table 1). Neutrophils were treated with LPS (5 µg·mL^−1^) or PMA (100 nM), which induce NADPH oxidase- and PKC-dependent NETosis [14–17]. PMA was chosen because this is the prototypical and most commonly used NET-inducer, as well as being profoundly effective at inducing NETs. A limitation of using PMA is that it is a chemical stimulant and considered by many as not physiologically relevant. To overcome this, we also induced NETs with LPS, which is a naturally occurring bacterially derived molecule. Although LPS is not directly associated with viral infection *per se*, it does model the additive effect of secondary bacterial infections, which are not uncommon in COVID-19 and which, *via* the effect on NETosis, may add to the inflammatory pathology seen in this disease. NET formation was measured by SYTOX™ Green staining of extracellular DNA [12, 16]. Compared with healthy control subjects, neutrophils from people with acute COVID-19 generated significantly more NETs in response to LPS and a similar amount of NETs in response to PMA (figure 1). Three patients were admitted to the intensive care unit (ITU) during our study (indicated as open squares in figure 1) and generated among the highest SYTOX™ Green values following PMA treatment. The increased capacity of neutrophils to undergo LPS-induced NETosis during the acute stage of COVID-19 adds to existing data suggesting this could be a key element of the dysregulated and deleterious inflammatory response in COVID-19.

### Dexamethasone does not modify elevated NETosis in hospitalised patients with COVID-19

**FIGURE 2:**
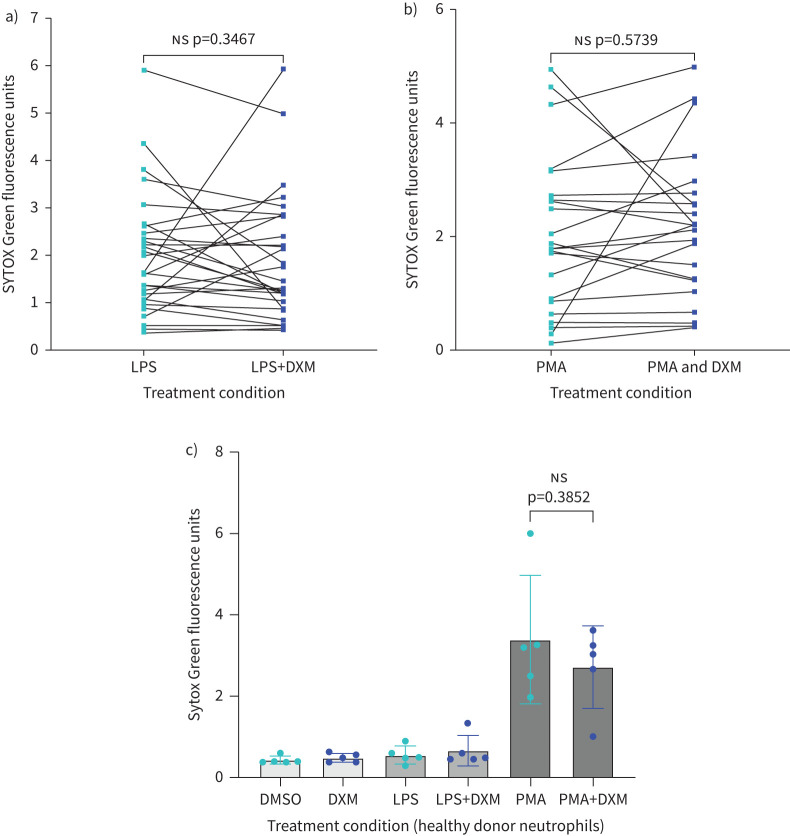
Dexamethasone (DXM) does not impact NETosis in neutrophils isolated from hospitalised coronavirus disease 2019 (COVID-19) patients or healthy donors. a and b) Neutrophils isolated from peripheral whole blood from hospitalised patients with COVID-19 were pre-incubated with DXM (10 µM), for 1 h (dark blue squares). Neutrophils were stimulated with LPS (5 µg·mL^−1^) (a, n=31) or phorbol myristate acetate (PMA) (100 nM) (b, n=23) for a further 3 h (pale blue squares). SYTOX Green was added and extracellular DNA (NETs) was quantified using a fluorescent plate reader. c) The assay was repeated using healthy donor neutrophils (pale blue circles) treated with dexamethasone as before (dark blue circles) (c, n=5). Data set (a) was normally distributed and a paired t-test was conducted. Data set (b) was not normally distributed and a Wilcoxon matched-pairs signed rank test was conducted. A one-way ANOVA with a Bonferroni's selected pairs post-test was completed for panel c where PMA alone was compared with PMA+DXM. Error bars represent sd.

Dexamethasone was the first therapy demonstrated to reduce COVID-19-associated mortality and was licensed for use in treating hospitalised COVID-19 patients requiring supplemental oxygen in September 2020 [18]. Dexamethasone is an anti-inflammatory drug and has previously been shown to reduce neutrophil recruitment and NETosis both in *in vitro* and *in vivo* murine models [19, 20]. We examined whether pre-treatment with dexamethasone (10 µM) for 1 h prior to stimulation with LPS or PMA, as before, impacted the NETosis response from COVID-19 neutrophils. There was no significant effect of dexamethasone on either LPS- or PMA-induced NETosis (figure 2a and b). However, 82% of COVID-19 patients in the study were receiving dexamethasone at time of sampling, meaning neutrophils analysed could have been previously exposed to the drug *in vivo*. The experiment was therefore repeated with healthy donor neutrophils, which were naïve to dexamethasone. While the LPS-induced NET response was low, as is typically seen with this concentration in healthy neutrophils, there was no impact of dexamethasone on either LPS- or PMA-induced NET formation using healthy donor neutrophils (figure 2c).

### The orally active inhibitor of PKC, ruboxistaurin, inhibits LPS-induced *ex vivo* NET formation in COVID-19

**FIGURE 3:**
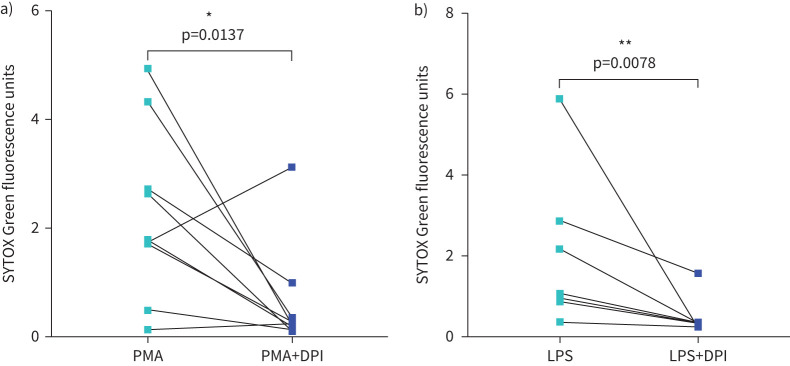
The reactive oxygen species (ROS) inhibitor diphenyleneiodonium (DPI) significantly reduces both phorbol myristate acetate (PMA)- and LPS-stimulated NET formation in neutrophils from acute coronavirus disease 2019 (COVID-19) patients. Neutrophils isolated from peripheral whole blood from hospitalised patients with COVID-19 were pre-incubated with ROS inhibitor, DPI (10 µM), for 1 h (dark blue squares). Neutrophils were stimulated with PMA (100 nM) (a, n=9) or LPS (5 µg·mL^−1^) (b, n=7) for a further 3 h (pale blue squares). SYTOX Green was added and extracellular DNA (NETs) was quantified using a fluorescent plate reader. Statistical analysis was performed by one-tailed Wilcoxon matched-pairs signed rank test, and significance values are as indicated. *: p<0.05; **: p<0.01.

**FIGURE 4:**
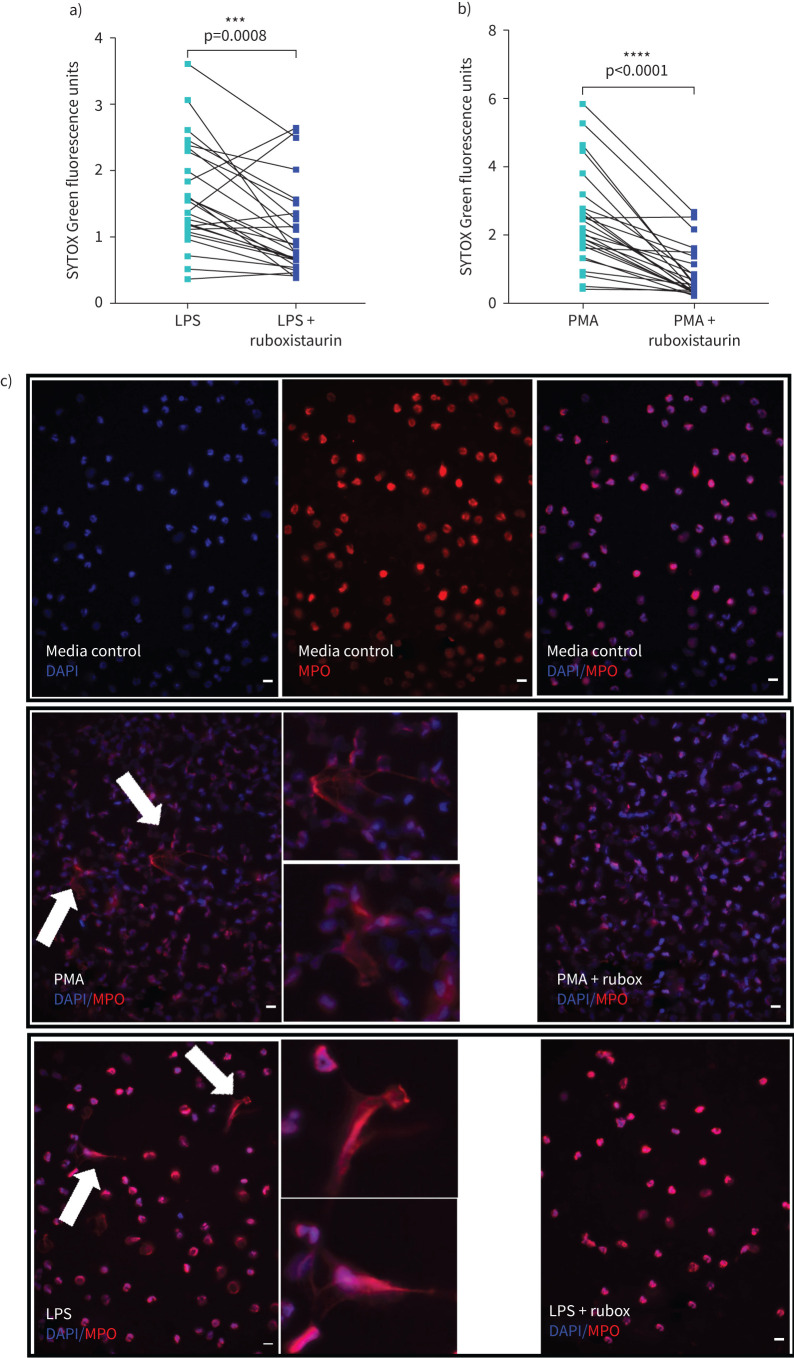
Ruboxistaurin significantly reduces both LPS- and phorbol myristate acetate (PMA)-stimulated NET formation in neutrophils from acute coronavirus disease 2019 (COVID-19) patients. Neutrophils isolated from peripheral whole blood from hospitalised patients with COVID-19 (a, n=26; b, n=28) were pre-incubated with ruboxistaurin (200 nM) for 1 h (dark blue squares). Neutrophils were stimulated with LPS (5 µg·mL^−1^) or PMA (100 nM) for 3 h (pale blue squares). SYTOX Green was added and extracellular DNA (NETs) was quantified using a fluorescent plate reader. Statistical analysis was performed by Wilcoxon matched-pairs signed rank test (a and b) and significance values are as indicated. c) COVID-19 patient derived neutrophils were seeded in IBIDI™ chamber wells and stimulated as described for panels a and b, plus media control. Neutrophils were stained for myeloperoxidase (MPO) and detected using Alexa Fluor 597 fluorochrome (red). DNA was visualised with ProLong™ Gold Antifade Mountant with DAPI (blue). Cells were viewed by fluorescence microscopy (40× magnification) and images are representative of three independent experiments. Fields of view were selected at random. Arrows indicate NETs (zoomed images show NET morphology). Scale bar=10 µm. ***: p<0.001; ****: p<0.0001.

NETosis can occur *via* ROS-dependent mechanisms, and we set out to determine whether this was the case in the context of COVID-19 [13, 21, 22]. We show both PMA- (figure 3a) and LPS- (figure 3b) induced NET formation in neutrophils from people with acute COVID-19 is significantly reduced by the NADPH oxidase inhibitor, DPI. PKC is a key signalling component of ROS-dependent NET formation [16]. Ruboxistaurin is an effective inhibitor of PKC-β, has completed phase III clinical trials for diabetic retinopathy and is well tolerated by patients [23]. We show for the first time that ruboxistaurin is a potent inhibitor of NET formation in COVID-19 neutrophils, significantly reducing both LPS- (figure 4a) and PMA- (figure 4b) induced NETs. During NETosis, neutrophils release DNA which is decorated with antimicrobial components including MPO [24]. We confirm biochemically and morphologically that neutrophils from patients with COVID-19 generate MPO-positive NETs in response to PMA and LPS and that fewer NETs are visualised in the presence of ruboxistaurin (figure 4c). To understand whether components of SARS-CoV-2 could directly induce NET formation that is amenable to inhibition by ruboxistaurin, we incubated neutrophils from healthy subjects (to exclude the possibility that neutrophils had previously been exposed to viral proteins *in vivo*) with purified SARS-CoV-2 nucleocapsid and spike proteins. Neither antigen induced NETs alone, nor in the presence of LPS, supporting the observation that SARS-CoV-2-mediated NETosis is dependent on viral replication (supplementary figure S1) [8, 13].

**FIGURE 5:**
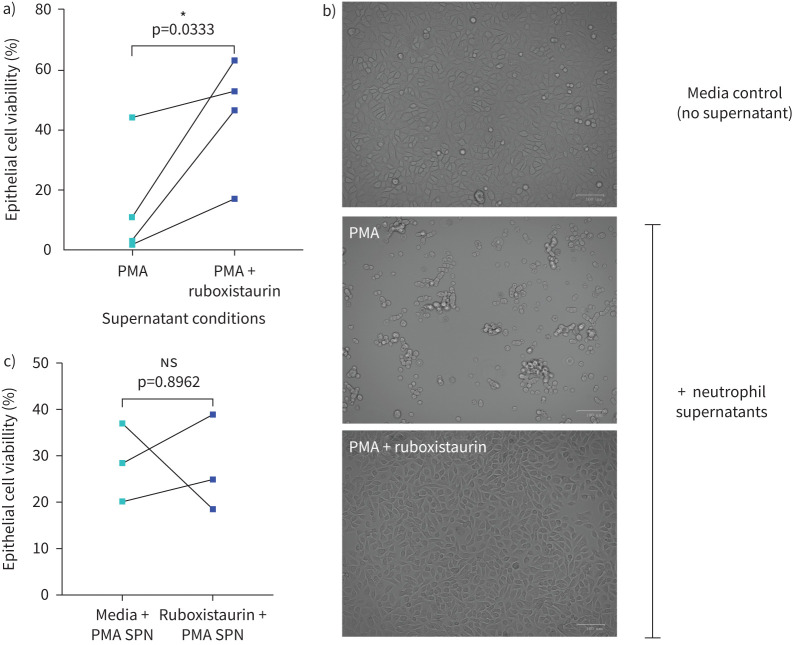
Ruboxistaurin reduces neutrophil supernatant-induced epithelial cell damage. a) Cell-free supernatants (SPNs) from neutrophils isolated from healthy donors stimulated with phorbol myristate acetate (PMA) (100 nM)±ruboxistaurin (200 nM) were added to confluent human bronchial epithelial cells (HBEC3-KT) at a 1:2 dilution. After 24 h HBEC3-KT-cell viability was assessed using CellTitre GloⓇ (n=4). b) Cell monolayers were imaged using the Zoe fluorescent-cell imager, using the brightfield setting and 20× objective lens. Images are representative of four donors, and fields of view were selected at random. Scale bar=100 µm. c) Epithelial cells were incubated with either media (light blue squares) or ruboxistaurin (200 nM) (dark blue squares) plus SPNs from neutrophils isolated from healthy donors stimulated with PMA (100 nM) for 24 h. Cell viability was assessed as above (n=3). Statistical analysis was performed by t-test, and significance values are as indicated. ns: nonsignificant. *: p<0.05.

NETs directly induce epithelial cell damage [8, 25]. Here we show that SPNs from PMA-treated neutrophils isolated from healthy volunteers induce death of HBEC3-KT cells, which is significantly reduced by ruboxistaurin (figure 5a). Rounding up and detachment of the monolayer was visible in HBEC3-KT cells cultured with SPNs from PMA-treated neutrophils, which was reduced in the presence of ruboxistaurin (figure 5b). To determine whether ruboxistaurin was having a direct effect on epithelial cells, we incubated cells with media or ruboxistaurin and added SPNs from PMA-treated neutrophils. Ruboxistaurin does not reduce epithelial cell death, suggesting its protective effect is *via* neutrophils and the reduction in NET formation (figure 5c). Since secondary infections are not uncommon in COVID-19, it is important not to compromise neutrophil microbicidal functions. To this end, we measured killing of the human pathogen *Staphylococcus aureus* by COVID-19 neutrophils and show ruboxistaurin had no effect on the ability of neutrophils to kill *S. aureus* (data not shown).

### Elevated NETosis in acute COVID-19 patients reduces over time but remains higher than in healthy controls

**FIGURE 6:**
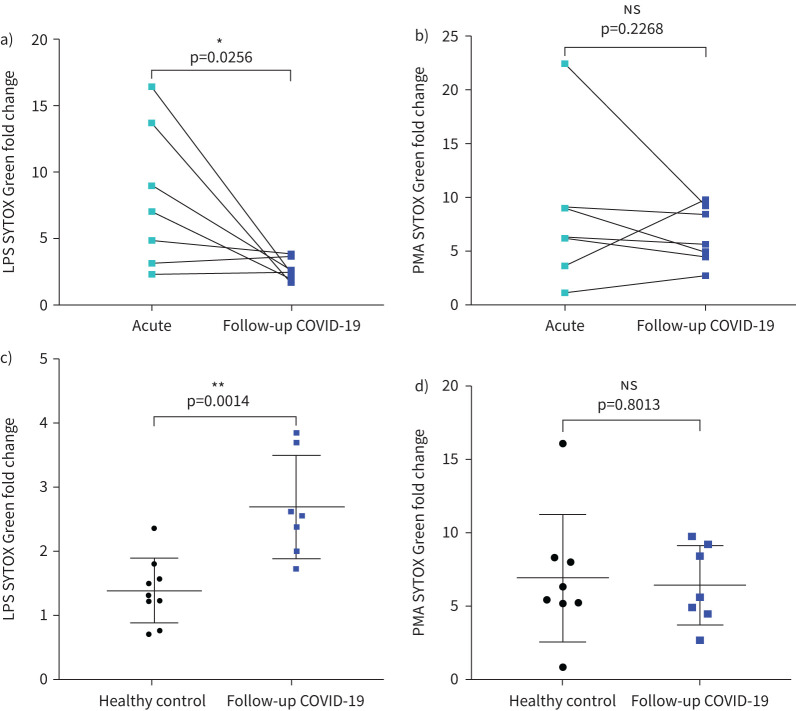
LPS-induced NETosis is reduced in follow-up coronavirus disease 2019 (COVID-19) patients but remains significantly higher than in healthy controls. Seven previously hospitalised patients with COVID-19 (dark blue squares), who were part of the acute COVID-19 cohort in figure 1a, returned to a follow-up clinic 3–4 months post-acute sampling (pale blue squares). Neutrophils were stimulated as previously described with LPS (5 µg·mL^−1^) (a) or phorbol myristate acetate (PMA) (100 nM) (b), and NET formation was quantified using SYTOX Green. To show linked data from individual patients at acute and follow-up time points, fold data were expressed by calculating fold change to DMSO control. Lines link values from the same patient (n=7). There was a significant reduction in LPS-induced NET formation at the follow-up time point but no difference in PMA-stimulated neutrophils. Follow-up data were also compared to healthy control data (black circles) (n=9), these control samples being also used in figure 1a, in response to LPS (c) or PMA (d). LPS-induced NETs were significantly higher in follow-up COVID-19 patients compared to healthy controls, but there was no difference in PMA-stimulated neutrophils. Statistical analysis was performed by a one-tailed paired t-test (a and b) and a two-tailed unpaired t-test (c and d), and significance values are as indicated. ns: nonsignificant. *: p<0.05; **: p<0.01.

Neutrophils have been shown to be reprogrammed during COVID-19, and we aimed to investigate whether the pro-NET phenotype observed during the acute stage persisted after infection [26]. To do this we studied a subset of seven individuals at follow-up clinics held 3–4 months following acute sampling (table 2). LPS-induced NETosis was significantly reduced at the follow-up time point (figure 6a) indicating a reduction in the pro-NETosis phenotype in this population. PMA-induced NETosis did not differ between the acute and follow-up time points (figure 6b). This is not unexpected since PMA is a potent inducer of NETs in healthy cells. In comparison to NETosis in healthy neutrophils however (previously shown in figure 1a), LPS-induced NET formation (figure 6c) but not PMA-induced NET formation (figure 6d) remained significantly elevated in neutrophils isolated at the follow-up time point.

## Discussion

Our findings show neutrophils isolated from patients with acute COVID-19 undergo significantly more LPS-induced NETosis than healthy control cells. Although LPS-induced NET formation significantly reduces in COVID-19 patients over time, levels at follow-up time points remained higher than in healthy control cells. We are the first to show that LPS-induced NETosis can be inhibited by ruboxistaurin *in vitro*, indicating a role for PKC-β in this pathway. This finding not only supports the importance of the PKC-β signalling pathway in neutrophils in COVID-19, but also reveals a potential therapeutic strategy for this disease.

Middleton *et al.* [6] demonstrated elevated baseline NET levels in neutrophils isolated from COVID-19 patients, which were not further increased by PMA. In contrast, we do not show elevated baseline (unstimulated) NET formation, which may reflect differences in disease severity, patient demographics, activation during the isolation procedure or sensitivity of the NET assay. Neutrophils from COVID-19 patients in our study robustly responded to PMA and generated NETs to levels comparable to healthy control cells. Interestingly, individuals with some of the greatest PMA-induced NET responses went on to require ITU support. Since this was a very small subgroup (n=3), more work is required to determine whether there is an association here.

While others have also shown increased NETosis in people with COVID-19 in response to PMA, a potent PKC activator, we are the first to show an increase in NETs in response to LPS, a receptor-driven neutrophil stimulator and typically less potent inducer of NETosis. Increased sensitivity to LPS-induced NETosis has implications in the case of secondary infections, and shows neutrophils are primed to increased NET formation to this, and therefore potentially other, proinflammatory stimuli [8]. The mechanism by which neutrophils from people with COVID-19 are more sensitive to undergoing NETosis is unclear. SARS-CoV-2 directly triggers NET formation [27] as does sera from COVID-19 patients [12]. In keeping with the work of others who show live, but not inactivated SARS-CoV-2 induces NETs [8, 13], we demonstrated that purified viral antigens did not induce NET formation. Furthermore, increased NET formation in isolated neutrophils *ex vivo* suggests this is not as a result of direct SARS-CoV-2 exposure and is more likely due to the neutrophils being in an activated and primed state and therefore being inherently more sensitive to NET stimuli. This is supported by other studies which describe neutrophil “hyperactivation” in COVID-19, whereby neutrophils are transcriptionally reprogrammed and which is a predictor of severe disease [28–30]. Furthermore, circulating neutrophils from critically ill COVID-19 patients have exaggerated ROS production which may contribute to increased NET production [31]. Although significantly less than at the acute stage of infection, LPS-induced NET formation remained higher in the subset of individuals who were resampled after 3–4 months, compared to healthy controls. This may suggest a pro-NETotic phenotype continues beyond the period of active SARS-CoV-2 infection, or could reflect pre-existing patient comorbidities in which increased NETosis is observed.

LPS is a weak inducer of NETs in healthy neutrophils compared with PMA, which in part explains why we do not see differences in PMA-induced NETs when comparing healthy control cells with neutrophils isolated from people with COVID-19 (regardless of the time point). It is possible that upregulation of the TLR4 receptor and/or downstream signalling components in COVID-19 are responsible for the increased sensitivity to LPS-induced (but not PMA-induced) NETs, as seen in monocytes [32]. SARS-CoV-2 spike protein activates TLR4 in neutrophil-like cells *in vitro*, and therefore has the potential to cause priming to subsequent exposure to LPS in circulating neutrophils, although whether sufficiently high levels of spike exist in the blood to allow this to occur is unknown [33].

A limitation of our study is around the demographics of the healthy control subjects compared to the patient cohort, the latter of whom are older, have more comorbidities and are receiving medications that may impact on neutrophil function (including dexamethasone). However, studying patients at 3–4 months post-acute sampling means participants serve as appropriate age- and comorbidity-matched controls and allow us to understand differences in neutrophil function at the acute stage of the disease.

Vaccination weakens the link between infection and critical illness, but vaccine breakthroughs are seen, particularly in the case of viral variants, such as the Omicron variant, which will continue to emerge [34]. It is therefore critical that we develop alternative and complementary strategies to prevent severe COVID-19 disease, and the innate immune response is an ideal target for this. Since NETs are known drivers of pathology in a number of diseases including but not limited to COVID-19, targeting NETosis is a logical therapeutic strategy for the future. A growing number of studies describe increased levels of NETosis in disease, as well as the deleterious role of NETs in driving inflammation, thrombosis and disease severity, but few have offered a solution. Our study indicates that ruboxistaurin could reduce NET formation and ultimately diminish airway inflammation and other events including microvascular thrombosis, and is a novel and promising therapeutic strategy for COVID-19 [6, 8]. Furthermore, our preliminary data demonstrates reducing NET formation with ruboxistaurin protects airway epithelial cells *in vitro*. Maintaining airway epithelial integrity could provide protection against secondary bacterial infection, which is important since secondary infection is a predictor of death in COVID-19 patients [35, 36]. Targeting NETosis in COVID-19 is a strategy shared by others in the field. Therapies inhibiting NET-associated protease activity (NCT04817332) and targeting the breakdown of NETs with DNases are also currently in clinical trials for COVID-19 (NCT04359654). However, ruboxistaurin has the advantage that it prevents NET formation by circulating neutrophils, rather than either modifying or disrupting NETs once they have been formed. Ruboxistaurin has been demonstrated to reduce NET formation in an *in vivo* mouse model and an *in vitro* study of healthy neutrophils, suggesting it has promise in targeting NETs in disease [16, 37]. Since phase 3 trials for diabetic retinopathy show ruboxistaurin is a well-tolerated inhibitor of PKC, we believe it could be relatively quick to translate to the clinic, providing a novel therapeutic pathway to treat neutrophil-mediated immunopathology in COVID-19 [23].

## Supplementary material

10.1183/23120541.00596-2021.Supp1 **Please note:** supplementary material is not edited by the Editorial Office, and is uploaded as it has been supplied by the author.
Supplementary material 00596-2021.SUPPLEMENT
